# Combining the effects of process design and pH for improved xylose conversion in high solid ethanol production from *Arundo donax*

**DOI:** 10.1186/s13568-014-0041-z

**Published:** 2014-05-01

**Authors:** Benny Palmqvist, Gunnar Lidén

**Affiliations:** 1 Department of Chemical Engineering, Lund University, Lund, SE-221 00, Sweden

**Keywords:** Bioethanol, High solids loading, Xylose fermentation, SSCF, Enzymatic hydrolysis

## Abstract

The impact of pH coupled to process design for the conversion of the energy crop *Arundo donax* to ethanol was assessed in the present study under industrially relevant solids loadings. Two main process strategies were investigated, i.e. the traditional simultaneous saccharification and co-fermentation (SSCF) and a HYBRID design, where a long high temperature enzymatic hydrolysis step was carried out prior to continued low temperature SSCF, keeping the same total reaction time. Since acetic acid was identified as the major inhibitor in the slurry, the scenarios were investigated under different fermentation pH in order to alleviate the inhibitory effect on, in particular, xylose conversion. The results show that, regardless of fermentation pH, a higher glucan conversion could be achieved with the HYBRID approach compared to SSCF. Furthermore, it was found that increasing the pH from 5.0 to 5.5 for the fermentation phase had a large positive effect on xylose consumption for both process designs, although the SSCF design was more favored. With the high sugar concentrations available at the start of fermentation during the HYBRID design, the ethanol yield was reduced in favor of cell growth and glycerol production. This finding was confirmed in shake flask fermentations where an increase in pH enhanced both glucose and xylose consumption, but also cell growth and cell yield with the overall effect being a reduced ethanol yield. In conclusion this resulted in similar overall ethanol yields at the different pH values for the HYBRID design, despite the improved xylose uptake, whereas a significant increase in overall ethanol yield was found with the SSCF design.

## Introduction

The lignocellulosic ethanol industry is moving from pilot scale to demonstration/full scale operation, which is evidenced by the construction of several production facilities worldwide (Balan et al. [2013]; Janssen et al. [2013]). Driven by this transition, research has been intensified towards problems related to high solid operation, an important factor for process economy (Humbird et al. [2010]; Macrelli et al. [2012]). By increasing the content of water insoluble solids (WIS) throughout the process a number of benefits can be gained, e.g. reduced water usage with lowered distillation and waste water treatment costs as a result. Benefits can also be gained by reductions in investment and production costs since equipment size and energy consumption can be reduced (Galbe et al. [2007]; Wingren et al. [2003]).

Looking at some of the larger plants in operation today, various agricultural residues (or in some cases dedicated energy crops) are used as raw material to a large extent. One example is Beta Renewables' full scale plant in Crescentino, Italy, which will convert wheat straw and *Arundo donax* (is a perennial cane used as a dedicated energy crop) to bioethanol, with biogas and bioelectricity as the main co-products. A common feature of these straw based raw materials is their relatively high content of the C5 sugar xylose (Wiselogel et al. [1996]), which makes good xylose conversion another high priority target in order to reach an economically feasible process (Sassner et al. [2008]). Today a number of *Saccharomyces cerevisiae* strains (the common workhorse in the bioethanol industry) have been genetically engineered in order to convert also xylose to ethanol. The main genetic modifications made are the insertion of either a bacterial xylose isomerase, or a fungal xylose reductase and xylitol dehydrogenase, together with over-expression of several genes in the pentose phosphate pathway (PPP) in order to convert xylose to xylulose and further on to ethanol through the PPP (Almeida et al. [2011]; Van Vleet and Jeffries [2009]; Hahn-Hägerdal et al. [2007]). Although impressive achievements have been accomplished with genetic and evolutionary engineering, glucose is still the preferred substrate over xylose. It has, however, previously been shown that xylose conversion can be increased with clever process design (Olofsson et al. [2010a]; Olofsson et al. [2008]; Olofsson et al. [2010b]).

As previously mentioned, working at high WIS content potentially improves process economy. However, high solid operation has also been shown to generally decrease the yields of both enzymatic hydrolysis and simultaneous saccharification and fermentation (SSF) (Kristensen et al. [2009b]). Two of the main issues when increasing the WIS loading are the dramatically increased viscosities as a result of the fibrous nature of the biomass (Knutsen and Liberatore [2009]; Roche et al. [2009]; Viamajala et al. [2009]; Wiman et al. [2011]) and the increased concentrations of biomass degradation products, e.g. hydroxymethylfurfural (HMF), furfural and acetic acid, which potentially inhibit the fermenting micro-organism (Almeida et al. [2011]). The formation of inhibitory degradations products, for example HMF and furfural, can be avoided by designing a mild pretreatment step. Acetic acid, in contrast, is inherent in the biomass material itself where acetyl groups are present on the xylan backbone. During pretreatment (and possibly enzymatic hydrolysis) the acetyl groups are released from the hemicellulose, hence forming acetic acid, which may inhibit the fermentation. It has been shown that the inhibitory effects can be decreased by operating at a higher pH-value, since it is the undissociated form of the acid that causes inhibition. The pKa value of acetic acid is 4.76, so large effects can be anticipated around a pH-value of 5. The positive effect has been shown to be particularly strong for xylose fermentation (Bellissimi et al. [2009]; Casey et al. [2010]).

The increased viscosities of the high solid slurries can create mixing problems in the reactors (Viamajala et al. [2010]) as well as problems in pumping of the slurry. Well mixed hydrolysis, and fermentation, processes are important in order to avoid temperature, pH and concentration gradients, since these could cause yield losses. One way to quickly reduce viscosity, and ease mixing, is the introduction of a high temperature hydrolysis step, commonly referred to as viscosity reduction (VR), or liquefaction (Jorgensen et al. [2007]), prior to the more traditional SSF concept. This HYBRID process design, where a high temperature hydrolysis is followed by a low temperature fermentation (and continued hydrolysis) of the whole remaining slurry is potentially a good process option. It allows (partly) independent optimization of the hydrolysis and fermentation steps, which is beneficial since the optimal temperature for the cellulose mixture typically is about 50˚C whereas the yeast grows optimally at temperatures around 30–35˚C. Furthermore, it may be beneficial to increase the fermentation pH above 5.0 (the typical optimum for enzymatic hydrolysis) in order to decrease the toxic effects of the hydrolyzate if high amounts of acetic acid are present. The improvement of commercially available enzyme mixtures (McMillan et al. [2011]) works towards favoring of this HYBRID process concept due to improved temperature stability and decreased end-product inhibition, as argued by Cannella and Jorgensen ([2013]). In the work by Cannella and Jorgensen, however, the added complexity of co-fermenting xylose and glucose to ethanol was not addressed. It is well-known that high glucose concentrations inhibit xylose uptake by the yeasts (Lee et al. [2002]; Saloheimo et al. [2007]). Therefore, in the case of co-fermentation of xylose, the SSCF process (with “C” indicating co-fermentation) has the advantage over a HYBRID process that you can keep a low, but non-zero, concentration of glucose in the media, which has been proven very beneficial for xylose consumption (Bertilsson et al. [2008]; Meinander et al. [1999]).

In this work, we assess how ethanol yields and xylose conversion are affected by the choice of process design at different pH levels. The raw material used in the study is pretreated *Arundo donax* at industrially relevant solids concentrations. Importantly, by analyzing fiber composition after each experiment the effects on the enzymatic hydrolysis, and the fermentation of both xylose and glucose could be assessed separately for the different scenarios. The effects of an increased pH at high acetic acid and sugar concentration were furthermore assessed separately in shake flask fermentations of fiber-free *Arundo donax* hydrolyzate.

## Materials and methods

### Raw materials

Steam pretreated *Arundo donax* slurry was kindly provided by Biochemtex S.p.A. Italia (Rivalta, Italy). The material was kept frozen until used. The fiber composition of the pretreated *Arundo donax* as well as the soluble sugars (Table 1) was determined according to NREL (National renewable energy laboratory) procedures (Sluiter et al. [2008a]; Sluiter et al. [2008b]). It can be noted from Table 1 that the major part of the soluble sugars still remains in oligomeric form after pretreatment and that the major inhibitor present is acetic acid. The WIS content of the material was determined to 22.5% by washing repeatedly with deionized water over filter paper (Whatman No.1).

**Table 1:** **Composition of the pretreated** ***Arundo donax*** **slurry**

**Solid composition (% of WIS)**		
	**Average**	**Std dev**
Glucan	48.2	0.2
Xylan	3.8	0.0
Galactan	n.d^a^	-
Arabinan	n.d	-
Mannan	n.d	-
Lignin	41.7	0.0
**Soluble components (g L**^**−1**^**)**		
**Sugars**	**Monomers**	**Total sugars (including monomers)**
Glucose	2.5	14.2
Xylose	4.0	18.4
Galactose	n.d	n.d
Arabinose	n.d	n.d
Mannose	n.d	n.d
**Inhibitors and degradation products**		
Acetic Acid	5.6	
HMF	n.d	
Furfural	0.2	

^a^n.d. = not detected, i.e. below detection limit.

The solid composition is based on wt-% of the WIS content and the soluble components are reported in g L^−1^ liquid. The WIS content of the pretreated material was measured to 22.5 wt-%.

### Cell cultivation

The recombinant xylose-fermenting strain *S. cerevisiae* TMB3400 (Wahlbom et al. [2003]) was used in all experiments. The yeast was produced by an initial pre-culture in shake flask, followed by an aerobic batch cultivation on glucose, and finally an aerobic fed-batch cultivation on *Arundo donax* hydrolyzate liquid, in order to improve inhibitor tolerance by adaptation as previously shown by Alkasrawi *et al.* (Alkasrawi et al. [2006]).

The yeast was inoculated (from agar plate) in 300 ml shake flasks (liquid volume of 100 ml) containing 16.5 g L^−1^ glucose, 7.5 g L^−1^ (NH_4_)_2_SO_4_, 3.5 g L^−1^ KH_2_PO_4_, 0.74 g L^−1^ MgSO_2_∙7H_2_O, trace metals and vitamins. The cells were grown for 24 h at 30°C and a starting pH of 5.2 in a rotary shaker at 180 rpm. Subsequently, aerobic batch cultivation was performed in a 2.5 L bioreactor (Biostat A, B. Braun Biotech International, Melsungen, Germany) at 30°C. The working volume was 0.7 L and the medium contained 20.0 g L^−1^ glucose, 20.0 g L^−1^ (NH_4_)_2_SO_4_, 10.0 g L^−1^ KH_2_PO_4_, 2.0 g L^−1^ MgSO_4_, 27.0 mL L^−1^ trace metal solution and 2.7 mL L^−1^ vitamin solution. The cultivation was initiated by adding 20.0 mL of the pre-culture to the bioreactor. The pH was maintained at 5.0 throughout the cultivation, by automatic addition of 3 M NaOH. The trace metal and vitamin solutions were prepared according to Taherzadeh *et al*. (Taherzadeh et al. [1996]). Aeration was maintained at 1.2 L min^−1^, and the stirrer speed was kept at 800 rpm. When the ethanol produced in the batch phase was depleted, the feeding of clarified hydrolyzate liquid (obtained by pressing the Arundo donax slurry) was initiated. 1.0 L of liquid (supplemented with 35 g/L glucose to ensure a sufficiently high final cell concentration) was fed to the reactor with an initial feed rate of 0.04 L h^−1^ which was increased linearly to 0.10 L h^−1^ during 16 h of cultivation. The aeration during the fed-batch phase was maintained at 1.5 L min^−1^, and the stirrer speed was kept at 800 rpm.

After cultivation, the cells were harvested by centrifugation in 700 mL flasks using a HERMLE Z 513 K centrifuge (HERMLE Labortechnik, Wehingen, Germany). The pellets were resuspended in 9 g L^−1^ NaCl-solution in order to obtain a cell suspension with a cell mass concentration of 80 g dry weight L^−1^. The time between cell harvest and initiation of the following SSCF/shake flask fermentation was no longer than 3 h.

### Hybrid SSCF experiments

All experiments were carried out under anaerobic conditions using 2.5 L bioreactors (Biostat A, B. Braun Biotech International, Melsungen, Germany) with an initial WIS content of 21% and a final working broth weight of 1.0 kg. The experiments were run for a total time of 96 h. SSCF experiments were compared to HYBRID experiments were a high-temperature hydrolysis step had been conducted for the first 48 hours (keeping a total time of 96 hours). During the enzymatic hydrolysis a temperature of 45˚C was maintained and when yeast was added (i.e. during the SSCF phase) the temperature was lowered and kept at 34˚C. The enzyme solution used was Cellic CTec 3 provided by Novozymes (Novozymes A/S, Bagsvaerd, Denmark) at a dose of 0.075 g enzyme solution g^−1^ glucan. The pH was maintained at either 5.0 or 5.5 throughout the fermentations by automatic addition of 4 M NaOH. The *Arundo donax* slurry was supplemented with 0.5 g L^−1^ NH_4_H_2_PO_4_, 0.025 g L^−1^ MgSO_4_ · 7H_2_O and 1.0 g L^−1^ yeast extract at the start of the fermentation phase. An initial yeast concentration of 3 g dry weight L^−1^ of the cultivated cells was used. In addition, a hydrolysis experiment was performed at 34˚C in order to see how well the material hydrolyzed at the lower SSCF temperature. All experiments were carried out in duplicates.

### Shake flask fermentations

100 mL shake flask fermentations were carried out in duplicates under anaerobic conditions in 300 mL Erlenmeyer flask. The medium used for the fermentations was the clarified liquid fraction obtained by separating the remaining solids (with vacuum filtration) after enzymatic hydrolysis of steam pretreated *Arundo donax*. The enzymatically hydrolyzed steam pretreated material was provided by Biochemtex and the separation of solids was carried out at Lund University. Two different acetic acid levels were investigate by first diluting the hydrolyzate slightly to achieve an acetic acid concentration of approximately 4 g L^−1^ and then supplementing one part of it with acetic acid to reach 8 g L^−1^. pH was set initially to 5.0, 5.5 and 6.0, respectively, for both acetate levels. Prior to fermentation the medium was autoclaved, to ensure sterile conditions, and supplemented with 0.5 g L^−1^ NH_4_H_2_PO_4_, 0.025 g L^−1^ MgSO_4_ · 7H_2_O and 1.0 g L^−1^ yeast extract (same supplements as in the hybrid SSCF experiments). The temperature was kept constant at 34˚C. An initial yeast concentration of 3 g dry weight L^−1^ of cultivated yeast was added to start the experiment. 2.5 mL liquid samples were taken repeatedly during 48 hours and analyzed for sugars and metabolites as well as pH and optical density.

### Analysis

HPLC was used for analysis of sugars and metabolites. Samples from the hydrolysis liquid were centrifuged (16,000 × g) in 2 mL eppendorf tubes at 14,000 rpm for 5 min. (Z 160 M, HERMLE Labortechnik, Wehingen, Germany). The supernatant was filtered through 0.2 μm filters, and stored at −20°C. The concentration of sugars, glycerol and xylitol were determined using a polymer column (Aminex HPX-87P, Bio-Rad Laboratories, München, Germany) at 85°C. MilliQ-water was used as eluent, with a flow rate of 0.6 mL min^−1^. Organic acids, furans and ethanol concentrations were determined with an Aminex HPX-87H column (Bio-Rad Laboratories, München, Germany) at 60°C. 50 mM H_2_SO_4_ was used as eluent, with a flow rate of 0.6 mL min^−1^. The sugars, acetate, glycerol, xylitol and ethanol were detected with a refractive index detector (Waters 2410; Waters, Milford, MA, USA) and HMF and furfural with a UV detector at a wavelength of 210 nm (Waters 2487; Waters, Milford, MA, USA). A larger sample volume was taken for the final (96 h) and liquid densities were determined (solids separated by centrifugation and filtration, as described above) by pippeting 0.500 ml on to an analytical scale to record the weight. Triplicates were performed with a standard deviation of less than 1%.

Optical density (OD) was measured with a spectrophotometer at 600 nm (Helios Gamma; Spectronic Camspec Ltd, Leeds, UK) in order to follow cell growth during the shake flask fermentations. The OD values were then correlated to cell dry-weight through a calibration curve obtained from dry-weight measurements and dilutions of the concentrated yeast suspension obtained after the cultivation procedure.

A one-way analysis of variance (ANOVA) was performed in MATLAB R2011b (Mathworks, Natick, USA) to established significant differences in fermentation yields and xylose uptake for both the reactor and shake flask experiments.

### Calculation of yields and carbon recovery

The standard assumptions when calculating glucose and ethanol yields are generally those of a constant volume and liquid density throughout the reaction. However, significant errors in yield calculations will result from using these assumptions during high solids operation (Kristensen et al. [2009a]; Zhu et al. [2011]). Therefore, in order to accurately estimate the hydrolysis yield in this study, we measured the WIS content and analyzed the solid composition according to standard NREL procedure (Sluiter et al. [2008b]) after each fermentation. Hence the hydrolysis yields (Y_Hyd_) could be calculated as the difference in total amount of glucan between start and end samples, according to Equation 1. The degree of xylan hydrolysis was calculated analogously.

In all following equations, m_reac_ denotes the total mass of the slurry, WIS the fraction of water insoluble solids in the total slurry (g/g), x_i_ the mass fraction of the respective polymer in the insoluble solids (g/g) and φ_i_ the molecular ratio of the polymer to its corresponding monomer. V_Liq_ denotes the volume of hydrolysate liquid (L) and ρ_Liq_ the corresponding density of the liquid (g/L). [i] denotes the concentration (g/L) of compound i, measured by HPLC, and M_i_ the c-mole mass of the compound. The short notations for the compounds are Glu (glucose), Xyl (xylose), Xyli (xylitol), Gly (glycerol), Cel (cellobiose), X (biomass) and EtOH (ethanol). The subscript tot, indicates the concentration determined by total sugar analysis for the respective sugars and subscript 0 and end indicates values at 0 and 96 hour respectively. The initial and final mass, m_reac_, will be slightly different due to the loss of carbon dioxide in the fermentation. The loss will be in the order of a few percent, and was in the calculations made here not compensated for.

(1) $$Y_{\mathrm{Hyd}}=\frac{m_{\mathrm{rea}c_{0}}\mathrm{WI}S_{0}X_{\mathrm{Gl}u_{0}}-m_{\mathrm{rea}c_{\mathrm{end}}}\mathrm{WI}S_{\mathrm{end}}X_{\mathrm{Gl}u_{\mathrm{end}}}}{m_{\mathrm{rea}c_{0}}\mathrm{WI}S_{0}X_{\mathrm{Gl}u_{0}}}$$

In order to further, calculate the ethanol yield based on measured HPLC concentrations, liquid densities were measured for both start and final samples (in addition to the WIS and glucan content). The fermentation yield, Y_EtOH_, (based on the amount of consumed glucose and xylose) could then be calculated on a mass basis according to Equation 2, and the technical ethanol yield, Y_EtOH,Tech_, (based on the total amount of added glucose and xylose) could be calculated according to Equation 3. Both equations are based on an equation previously presented by Kristensen *et al.* (Kristensen et al. [2009a]).

(2) $$\begin{array}{ll} Y_{\mathrm{EtOH}}= & \frac{{\left[\mathrm{EtOH}\right]}_{\mathrm{En}d^{V}\mathrm{Li}q_{\mathrm{End}}}}{m_{\mathrm{rea}c_{0}}\mathrm{WI}S_{0}\left(\varphi_{\mathrm{Glu}}X_{\mathrm{Glu}0}+\varphi_{\mathrm{Xyl}}X_{\mathrm{Xyl}0}\right)-m_{\mathrm{rea}c_{\mathrm{end}}}\mathrm{WI}S_{\mathrm{end}}\left(\varphi_{\mathrm{Glu}}X_{\mathrm{Gl}u_{\mathrm{end}}}+\varphi_{\mathrm{Xyl}}X_{\mathrm{Xy}l_{\mathrm{end}}}\right)+\left({\left[\mathrm{Gl}u_{\mathrm{tot}}\right]}_{0}+{\left[\mathrm{Xy}l_{\mathrm{tot}}\right]}_{0}\right)V_{\mathrm{Li}q_{0}}-\left({\left[\mathrm{Glu}\right]}_{\mathrm{End}}+{\left[\mathrm{Xyl}\right]}_{\mathrm{End}}\right)V_{\mathrm{Li}q_{\mathrm{End}}}} \end{array}$$

(3) $$Y_{\mathrm{EtO}H_{\mathrm{tech}}}=\frac{{\left[\mathrm{EtOH}\right]}_{\mathrm{En}d^{V}\mathrm{Li}q_{\mathrm{End}}}}{m_{\mathrm{rea}c_{0}}\mathrm{WI}S_{0}\left(\varphi_{\mathrm{Glu}}X_{\mathrm{Glu}0}+\varphi_{\mathrm{Xyl}}X_{\mathrm{xylo}}\right)+\left({\left[\mathrm{Gl}u_{\mathrm{tot}}\right]}_{0}+{\left[\mathrm{Xy}l_{\mathrm{tot}}\right]}_{0}\right)V_{\mathrm{Li}q_{0}}}$$

The liquid volume was calculated according to Equation 4.

(4) $$V_{\mathrm{Liq}}=\frac{m_{\mathrm{reac}}\left(1-\mathrm{WIS}\right)}{\rho_{\mathrm{liq}}}$$

A calculation of the carbon recovery (CR), on c-mole basis, was done for both the hybrid SSCF and shake flask experiments in order to see if all major products had been accounted for. Since carbon dioxide was not measured the amount was instead estimated based on stoichiometry from the ethanol production, hence the factor 1.5 (c-mole c-mole^−1^) in Equation 5. Furthermore, due to (well established) difficulties in estimating cell dry weight in fiber slurries, *biomass was not included* in the recovery calculations for the SSCF/HYBRID experiments, i.e. part of the missing carbon will be biomass. Biomass was, however, included in carbon recovery for the shake flask experiments.

(5) $$\mathrm{CR}=\frac{\left(\frac{1.5{\left[\mathrm{EtOH}\right]}_{\mathrm{End}}}{M_{\mathrm{EtOH}}}+\frac{{\left[\mathrm{Gly}\right]}_{\mathrm{End}}}{M_{\mathrm{Gly}}}+\frac{{\left[\mathrm{Xyl}\right]}_{\mathrm{End}}}{M_{\mathrm{Xyl}}}+\frac{{\left[\mathrm{Xyli}\right]}_{\mathrm{End}}}{M_{\mathrm{Xyli}}}+\frac{{\left[\mathrm{Cel}\right]}_{\mathrm{End}}}{M_{\mathrm{Cel}}}+\frac{\left[\mathrm{Gl}u_{\mathrm{End}}\right]}{M_{\mathrm{Glu}}}+\frac{{\left[X\right]}_{\mathrm{End}}}{M_{X}}\right)V_{\mathrm{Li}q_{\mathrm{End}}}+m_{\mathrm{rea}c_{\mathrm{end}}}\mathrm{WI}S_{\mathrm{end}}\left(\frac{\varphi_{\mathrm{Glu}}X_{\mathrm{Gl}u_{\mathrm{End}}}}{M_{\mathrm{Glu}}}+\frac{\varphi_{\mathrm{xyl}}X_{\mathrm{Xy}l_{\mathrm{End}}}}{M_{\mathrm{Xyl}}}\right)}{m_{\mathrm{rea}c_{0}}\mathrm{WI}S_{0}\left(\frac{\varphi_{\mathrm{Glu}}X_{\mathrm{Glu}0}}{M_{\mathrm{Glu}}}+\frac{\varphi_{\mathrm{xyl}}X_{\mathrm{Xyl}0}}{M_{\mathrm{Xyl}}}\right)+\left(\frac{{\left[\mathrm{Gl}u_{\mathrm{tot}}\right]}_{0}}{M_{\mathrm{Glu}}}+\frac{{\left[\mathrm{Xy}l_{\mathrm{tot}}\right]}_{0}}{M_{\mathrm{Xyl}}}+\frac{{\left[X\right]}_{0}}{M_{X}}\right)V_{\mathrm{Li}q_{0}}}$$

## Results

Experiments were made to compare the standard SSCF strategy to a HYBRID scenario in which a 48 hour high temperature hydrolysis step was conducted prior to continued hydrolysis and fermentation, at lower temperature, keeping a total process time of 96 hours. The strategies were investigated at two different pH levels, in order to elucidate how the inhibitory effect of the present acetic acid affected especially xylose consumption during the different process designs (Table 2). Post-fermentation material analyses of the residual solids were furthermore conducted for all the experiments and with these analysis it was possible to calculate the amount of released sugars and hence also the amount consumed by the yeast during fermentation. Based on this, the hydrolysis performance could be separated from the fermentation performance and be assessed individually for the different process designs. Furthermore the analysis allowed for the calculation of carbon recovery for each experiment by taking volume and density changes during the process into account (Eq 5). The carbon balance could be closed around 95% for the enzymatic hydrolysis experiment, whereas for the HYBRID/SSCF experiments where biomass could not be measured (and CO2 only estimated), the carbon recovery was slightly reduced, to around 90% (Table 2). This indicates that all major compounds have been considered.

**Table 2:** Hydrolysis and fermentation performance for the different process designs

	**SSCF**	**HYBRID**	**SSCF**	**HYBRID**	**Hydrolysis**
	**pH 5.0**	**pH 5.0**	**pH 5.5**	**pH 5.5**	**34°C**
**Hydrolysis performance**					
Degree of glucan hydrolysis (%)	49.4 ± 0.8	54.2 ± 0.5	50.9 ± 3.8	54.0 ± 1.1	45.2 ± 0.3
Degree of xylan hydrolysis (%)	35.9 ± 2.7	43.0 ± 1.8	38.1 ± 5.3	43.3 ± 0.5	31.1 ± 0.6
**Fermentation performance**					
Ethanol yield (g/g consumed sugars)	0.42 ± 0.01^A^	0.43 ± 0.00 ^A^	0.40 ± 0.02 ^A^	0.39 ± 0.02 ^A^	-
Ethanol yield (% of theoretical)	81.7 ± 1.9	85.0 ± 0.1	79.3 ± 4.7	76.4 ± 3.5	-
Glycerol yield (g/g consumed sugars)	0.015 ± 0.002	0.031 ± 0.004	0.028 ± 0.004	0.039 ± 0.003	
Consumed xylose [%]	40.2 ± 3.8	33.7 ± 9.2	78.3 ± 2.0	55.8 ± 5.5	-
Xylitol production (% of consumed xylose)	32.0 ± 0.1	15.9 ± 0.2	25.5 ± 0.2	19.2 ± 0.4	-
**Technical ethanol yield [%]**	40.3 ± 0.7 ^A^	45.2 ± 1.3 ^B^	44.4 ± 0.4 ^B^	42.7 ± 0.8 ^A B^	-
Calculated carbon recovery^*^(excluding cell growth)	0.93 ± 0.01	0.94 ± 0.00	0.92 ± 0.03	0.90 ± 0.02	0.96 ± 0.00

^*^Carbon recovery calculated according to Eq. 5 in material and methods, with CO_2_ estimated based on ethanol production.

Yields are based on the final (96 hour) values, and standard deviations are based on duplicate experiments. All statistically compared mean values are denoted with one or several letters (A, B). Values labeled with the same letter are not significantly different at a confidence level of 95%.

### The effects of changing pH at different process designs

**Figure 1:**
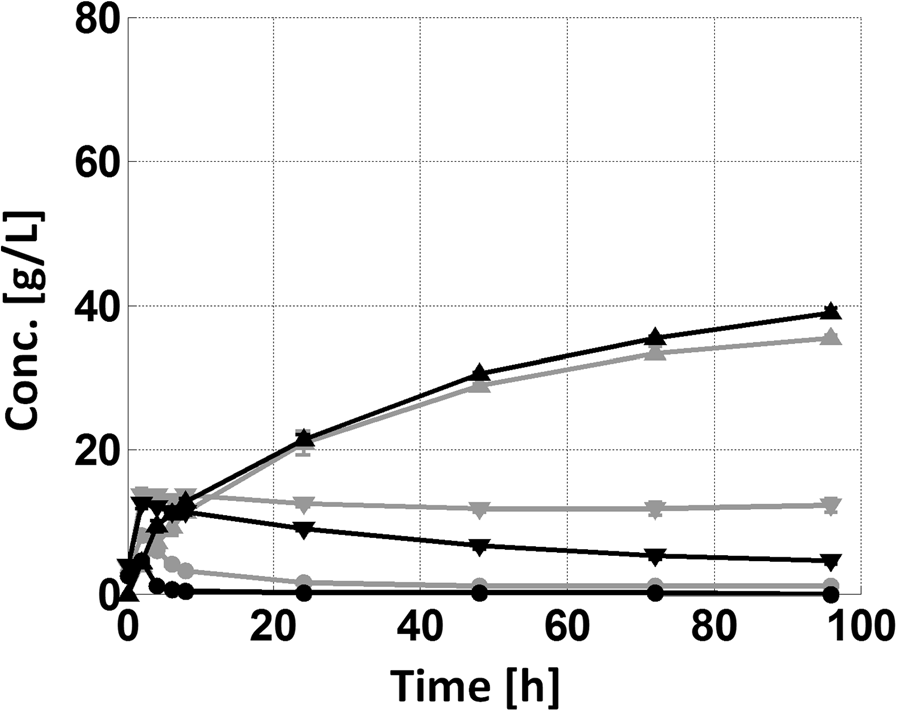
**Concentration profiles throughout the SSCF experiments at pH 5.0 (grey) and 5.5 (black).** Glucose (●), xylose (▼) and ethanol (▲). The error bars represent standard deviation of duplicate experiments.

**Figure 2:**
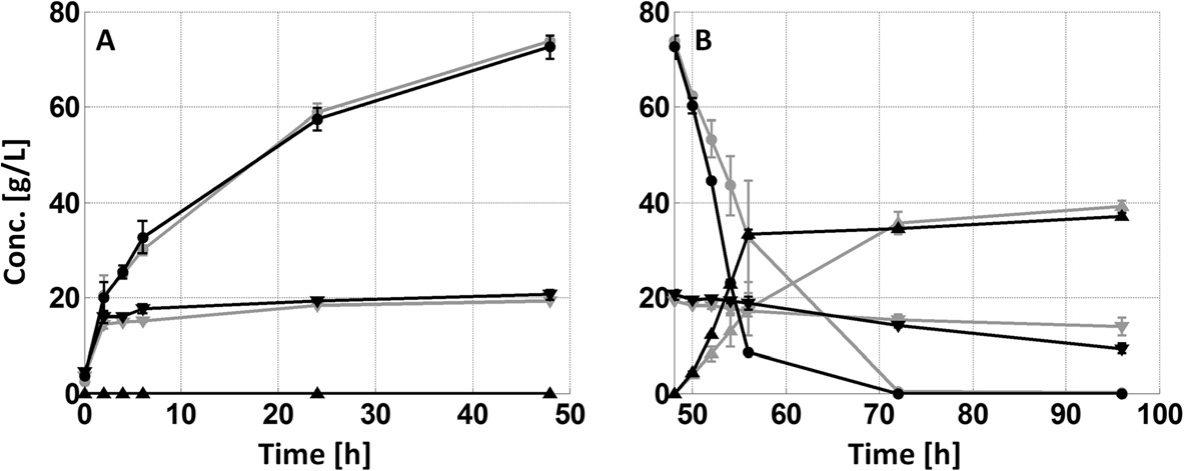
**Concentration profiles throughout the HYBRID process design at pH 5.0 (grey) and 5.5 (black). A)** The 48 hour high temperature hydrolysis. **B)** The SSCF phase during the final 48 hours. Glucose (●), xylose (▼) and ethanol (▲). The error bars represent standard deviation of duplicate experiments.

Increasing the pH from 5.0 to 5.5 during the SSCF design resulted in approximately a 10% increase in final ethanol concentration – i.e. from 35 to 39 g L^−1^ (Figure 1). Likewise, a significant increase in xylose consumption was achieved at the higher pH level. It should be noted that the xylose concentration in Figure 1 (and Figure 2) is the pseudo-steady state concentration generated by simultaneous xylan hydrolysis and xylose fermentation. Post material analysis however showed no significant difference in hydrolysis degree between the different pH levels (Table 2), hence the consumption can be a assumed to have increased. The post-fermentation material analysis did not show any significant difference in the degree of hydrolysis (Table 2), suggesting that the increased ethanol yield is a result of increased xylose fermentation. In contrast, when the pH in the fermentation phase of the HYBRID process was increased, no corresponding increase in ethanol could be observed (Figure 2), despite enhanced xylose consumption. Since no significant differences in hydrolysis performance was observed (Figure 2A and Table 2), this points towards a decrease in fermentation yield (although no statistically significant difference was found) at the higher pH. A potential yield reduction could be related to the faster initial fermentation rate observed at the less inhibiting conditions at the higher pH (Figure 2B). At less inhibiting conditions, cell growth and associated glycerol production is favored, which typically reduces the ethanol yield. Cell growth could not be measured in the slurries, but an increase was indicated by the higher glycerol production at the higher pH level (Table 2).

Furthermore, the degree of hydrolysis was higher in the HYBRID process design in comparison to the SSCF case. As a reference to the SSCF experiment a 96 hour enzymatic hydrolysis experiment was run at 34˚C. As expected, a very significant temperature effect on the enzymatic hydrolysis was found and a similar sugar release was obtained after a 48 hour hydrolysis at 45˚C as after 96 h at 34˚C (data not shown). It was also found that the overall glucan conversion was increased in SSCF compared to an enzymatic hydrolysis at the same temperature, indicating end product inhibition (Table 2).

### Fermentation at different pH and acetic acid concentrations

**Figure 3:**
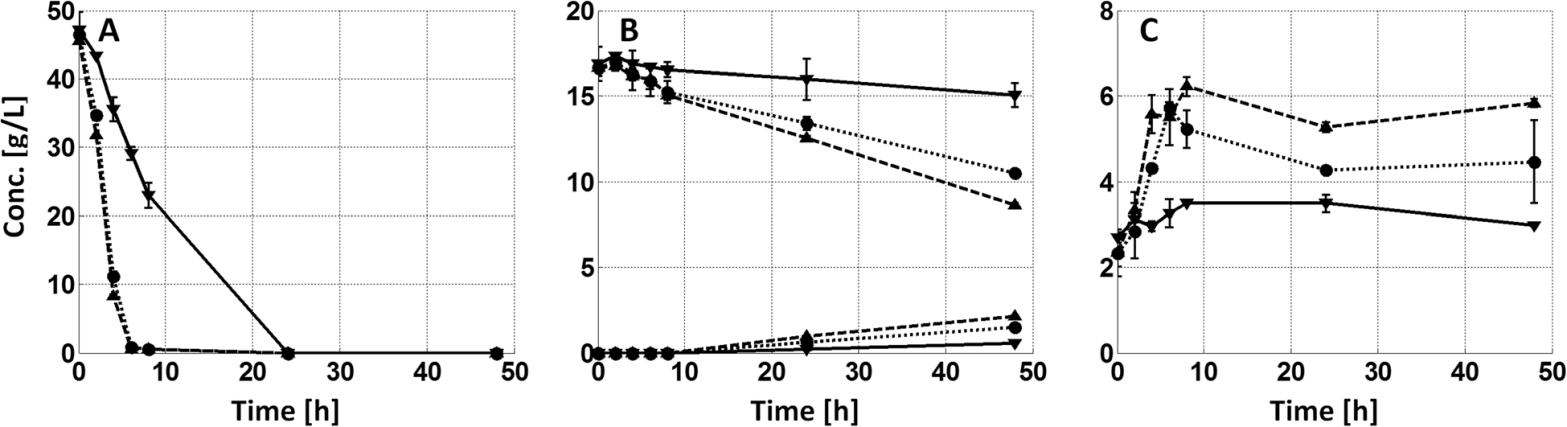
**Concentration profiles for glucose consumption (A), xylose consumption/xylitol production (B) and cell growth (C) during shake flask fermentations with 8 g L**^**−1**^**acetic acid at pH 5.0 (▼), 5.5 (●) and 6.0 (▲).** The error bars represent standard deviation of duplicate experiments.

Shake flask fermentations were set up in order to further evaluate the effects of pH on acetic acid inhibition at two different acetic acid concentrations (4 and 8 g L^−1^) during fermentation of hydrolyzate liquid. Three different pH levels (5.0, 5.5 and 6.0) were used, resulting in a set of 6 different conditions. The xylose consumption was clearly enhanced by increased pH, regardless of acetic level (Table 3). However, it was also seen that the ethanol yield (based on consumed sugars) was reduced at the less inhibiting conditions, i.e. high pH and low acetic acid concentration. By measuring OD it could be confirmed that cell growth was indeed promoted together with enhanced glucose consumption when increasing pH at high acetic acid concentrations (Figure 3). However, under milder acetic acid conditions, i.e. 4 g L^−1^, the effect of increasing pH above 5.0 was not as evident on cell growth (data not shown). One need also keep in mind that these experiments were made without fibers.

**Table 3:** Summary of the fermentation performance during the shake flask fermentations

	**4 g L**^**−1**^**acetic acid**		
**pH**	**5.0**	**5.5**	**6.0**
**Ethanol yield (g/g consumed sugars)**	0.44 ± 0.01 ^A^	0.43 ± 0.00 ^A B^	0.40 ± 0.02^C^
**Ethanol yield (% of theoretical)**	85.5 ± 1.2	83.8 ± 0.6	78.5 ± 3.6
**Glycerol yield (g/g consumed sugars)**	0.043 ± 0.007	0.042 ± 0.002	0.045 ± 0.004
**Consumed xylose (%)**	27.4 ± 1.9^C^	38.7 ± 2.5 ^A^	50.2 ± 1.2 ^B^
**Xylitol production (% of consumed xylose)**	23.9 ± 0.2	25.2 ± 4.0	28.5 ± 2.6
**Calculated carbon recovery**^**a**^**(including cell growth)**	0.99 ± 0.00	0.99 ± 0.01	0.97 ± 0.04
	**8 g L**^**−1**^**acetic acid**		
**pH**	**5.0**	**5.5**	**6.0**
**Ethanol yield (g/g consumed sugars)**	0.43 ± 0.03 ^A D^	0.43 ± 0.01 ^A D^	0.42 ± 0.01 ^B C D^
**Ethanol yield (% of theoretical)**	84.9 ± 5.4	84.5 ± 1.9	82.3 ± 0.9
**Glycerol yield (g/g consumed sugars)**	0.039 ± 0.009	0.042 ± 0.003	0.046 ± 0.000
**Consumed xylose (%)**	10.7 ± 1.2 ^D^	37.0 ± 0.6 ^A^	48.0 ± 2.0 ^B^
**Xylitol production (% of consumed xylose)**	32.1 ± 7.8	23.8 ± 0.7	27.2 ± 2.0
Calculated carbon recovery^*^(including measured cell growth)	0.95 ± 0.04	0.99 ± 0.01	1.01 ± 0.02

^*^Carbon recovery calculated according to Eq. 5 in material and methods, with CO_2_ estimated based on ethanol production.

Yields are based on the final (48 hour) values, and standard deviations are based on duplicate experiments. All statistically compared mean values are denoted with one or several letters (A, B, C, D). Values labeled with the same letter are not significantly different at a confidence level of 95%. Note that in this table, yields for all six set-ups are compared.

## Discussion

The present work showed that the HYBRID design, i.e. a high temperature enzymatic hydrolysis step prior to fermentation, gave a higher overall glucan conversion compared to a 96 hours SSCF process (Table 2). This is well in-line with results reported by Cannella and Jorgensen for pretreated wheat straw (Cannella and Jørgensen [2013]), and supports their claim that commercial enzyme mixtures available today are less end-product inhibited and exhibit improved long time temperature stability. It deserves to be pointed out, that the advantage comes from the high temperature for enzymatic hydrolysis. If SSCF and pure hydrolysis is compared at the same (permissible) temperature, e.g. 34˚C as done in this study, a better glucan conversion is indeed obtained for the SSCF case (Table 2–first and last columns). This confirms that SSCF most likely still gives a reduced end-product inhibition of the enzymes. However, this decreased end-product inhibition does not outweigh the enhanced enzymatic activity at the higher temperatures, which resulted in a better overall glucan conversion in the HYBRID process design (Table 2).

With respect to fermentation, the fundamental difference between the two process designs is the availability of glucose throughout the fermentation. During the HYBRID design, a large fraction of the total amount of both glucose and xylose that are to be fermented to ethanol are present from the start of the fermentation, whereas during SSCF the glucose is slowly being released from the fibers throughout the process, hence limiting the fermentation rate. One observed effect of this difference in available glucose was that xylose consumption was better with the SSCF design in comparison to the HYBRID process, most likely since high initial glucose levels are known to inhibit xylose uptake (Lee et al. [2002]; Saloheimo et al. [2007]). The effect on xylose consumption from process design was, however, not as large as when increasing the fermentation pH. A higher pH was found to clearly reduce the inhibitory effect of the acetic acid, as previously shown by for example Casey et al. ([2010]). An increased pH significantly enhanced xylose consumption for both process designs, although the SSCF design was more favored than the HYBRID design (Table 2). The strong correlation between xylose utilization and pH in the presence of acetic acid was furthermore confirmed in shake flask fermentations (Figure 3B and Table 3).

The increase in pH for the HYBRID design resulted not only in improved xylose consumption, but also the glucose uptake rates were significantly improved at the less inhibiting conditions (Figure 2). Coupled to the increased sugar uptake rate, a reduced fermentation yield was observed together with increased glycerol production, potentially indicating increased cell growth. Since cell growth is very difficult to quantify in fiber suspensions, shake flask fermentations where carried out on fiber free hydrolyzate where it could be concluded that an overall faster glucose consumption at less inhibiting conditions was correlated to enhanced cell growth (Figure 3). This agrees well with previous studies on both pure glucose and mixed sugar fermentations with other strains of *S. cerevisiae*, where ethanol yields were reduced at increased pH in the presence of acetic acid as a consequence of improved anaerobic cell growth (and associated glycerol production) (Casey et al. [2010]; Taherzadeh et al. [1997]). However, for the SSCF experiment the fermentation yield seemed less affected compared to the HYBRID design, possibly since the glucose uptake rate was limited by the rate of hydrolysis.

## Conclusion

In conclusion, this study shows that the impact of an increased pH on the overall ethanol yield is not necessarily in agreement with an a priori expectation, but will depend on the process design. The overall ethanol yield increased significantly for the SSCF strategy at increased pH, whereas no significant difference (or even a slight reduction in yield) was observed with the HYBRID strategy. This can be explained by the difference in available sugars during the fermentation. The high sugar concentrations in the HYBRID design resulted in both a reduced fermentation yield at the higher pH as well as a hampered xylose consumption in comparison to the SSCF strategy.

## Competing interests

The authors declare that they have no competing interests.
